# Use of phage display technology for the selection of monoclonal antibodies in allergen research and diagnostics

**DOI:** 10.1186/2045-7022-4-S2-O9

**Published:** 2014-03-17

**Authors:** Marjolein Vandekerckhove, Bart Van Droogenbroeck, Marc Deloose, Hilde Lapeere, Gevaert Philippe

**Affiliations:** 1 ILVO - Institute for Agricultural and Fisheries Research, Technology and Food Science Unit, Merelbeke, Belgium; 2 Institute for Agricultural and Fisheries Research, Technology and Food Science Unit, Merelbeke, Belgium; 3 UZ Ghent, 3UZ Ghent, Department of Dermatology, Ghent, Belgium; 4 UZ Ghent, Department of Oto-rhino-laryngology, Ghent, Belgium

## Background

Food allergies constitutes an important global health issue for the allergic consumers, the food industry and de medical health care. Since no treatment for food allergy is yet available, the allergic patient should completely avoid the threatening allergens from his diet. This is not easy to achieve in practice because the allergic consumer can already have an allergic reaction after eating traces of relevant allergens due to cross-contamination. This is the reason why many food manufactures make more and more use of precautionary labels such as 'may contain traces of...' To deal with this, there is an urgent need for analytical methods than can be used in food industries which should be highly specific and sensitive, detecting traces of allergens and be rapid, robust, reliable and cost-effective. For this moment, immunoassays are the most preferred method but there are only a limited number validated for only a few food allergens so research in this regard is still needed. Which allergenic compounds to detect and their change in allergenic potential during food processing are examples of important issues to be taken into consideration.

The main focus of this project is the generation of relevant monoclonal recombinant antibodies against hazelnuts which are patient-dependent and/or highly specific. Hazelnuts are widely used in the food industry and belong to one of the most important food allergens.

## Methods

Purified antigens were received from the EAACI group in Vienna (Cor a 1 and Cor a 8). First the purified antigens were used to generate highly specific nanobodies (VIB Nanobody Service Facility-VUB, Brussels) which still need to be further validated. These antibody fragments are interesting because of their high target specificity and --affinity, ease of manufacturing and excellent stability. In a second approach recombinant antibodies will be developed starting from the genetic information present in the blood of clinically characterized hazelnut allergic patients (scFv-fragments).

## Results

In the nanobody approach, the VHH-library was prepared (library size = 10^8 ) and selective panning rounds are currently being carried out. For the second approach, Cor a 1 and Cor a 8 patients were selected. Currently blood collection, mRNA isolation and cDNA preparation are planned.

## Conclusion

We believe that this approach can result in different antibody fragments valuable for a specific and sensitive detection of food allergens.

